# Different Correlates of COVID-19-Related Adherent and Dysfunctional Safety Behavior

**DOI:** 10.3389/fpubh.2020.625664

**Published:** 2021-01-13

**Authors:** Benjamin Weismüller, Adam Schweda, Nora Dörrie, Venja Musche, Madeleine Fink, Hannah Kohler, Eva-Maria Skoda, Martin Teufel, Alexander Bäuerle

**Affiliations:** Clinic for Psychosomatic Medicine and Psychotherapy, LVR-University Hospital Essen, University of Duisburg-Essen, Essen, Germany

**Keywords:** COVID-19, safety behavior, fear & anxiety, mental health, trust in government, subjective level of information, panic buying

## Abstract

**Introduction:** Safety behaviors are key elements in reducing the spread of the COVID-19 virus, but have also assumed excessive proportions in form of panic buying groceries. This raises the question whether these behaviors are independent or related to each other. Adherent safety behavior including increased hygiene and physical distancing appears inherently adherent and prosocial, while dysfunctional safety behavior such as panic buying most probably emerges from other motives and contextual variables.

**Methods:** Data from 15,308 participants collected from March 10 to May 4, 2020, during the COVID-19 acute period in Germany, was analyzed to assess whether adherent and dysfunctional safety behavior are predicted by the same or divergent variables. Two multiple regression models are presented including various sociodemographic, trait, attitudinal, and COVID-19-specific variables as predictors.

**Results:** Some variables similarly predict both, adherent and dysfunctional safety behavior. Yet, adherent safety behavior is stronger predicted by COVID-19-related fear than generalized anxiety, while a trend toward a reverse pattern emerged for dysfunctional safety behavior. Adherent safety behavior was also related to higher trust in governmental actions to face COVID-19, subjective level of information, as well as use of public media and TV to remain informed on COVID-19. Higher age was related to dysfunctional, but not adherent safety behavior. Respondents living in rural communities report more adherent safety behavior than urban dwellers.

**Discussion:** Divergent psychological variables underlie adherent and dysfunctional safety behavior. This hints toward a theoretical separation with practical relevance in behavioral engineering and public health campaigning.

## Introduction

In the very early days of the COVID-19 pandemic outbreak, fear and anxiety rapidly spread across the population in Germany (1–3). In turn, people started to hoard toilet paper and canned foods or even stole disinfectants from hospitals. To curtail infection rates, governmental authorities announced contact prohibitions, lockdowns, and most prominently mandatory mask-wearing. Some safety behaviors like hand-washing and physical distancing are adherent and actively prescribed by government authorities. An adherence to such safety behaviors is socially desirable and requires individuals to incur an immediate cost for the sake of society. However, panic buying and hoarding are rather egoistic behaviors as resources are limited and deprive others of them. Hence, while fear is spreading across the population (2), motives to wash hands seem to differ from motives to panic buy and hoard. Higher vulnerabilities to the virus caused by, e.g., cancer or chronic somatic diseases, but also mental illnesses, can increase fear and anxiety, which eventually leads to some safety behavior (4–6). The current strongly increasing numbers of new infections during the “second wave” on one hand, and political demonstrations against COVID-19 protection-regulations on the other hand, indicate the strong necessity to understand underlying motives for distinct safety behaviors.

Recent research on the behavioral-psychological impact of COVID-19 reveals the prevalence of safety behaviors like panic buying strongly increased since the onset of political measures during the COVID-19 crisis (3, 7–9). Especially panic buying has recently been a matter of strong interest for the public, the government, and research. Arafat et al. (10) showed that ~80% of the media reports on COVID-19 focused on the impact and causes of panic buying. Over half of these reports showed images of empty shelves to illustrate the topic. Furthermore, a quarter highlighted the rumor about panic buying and the remedial measures. Investigating the causes for panic buying, Arafat et al. (7) showed that a sense of scarcity seems to be the most prominent factor alongside an increased demand and importance of a product and the anticipation of rising prices facilitated this effect. Furthermore, the authors reported rumors, safety-seeking behavior, uncertainty, anxiety reduction, and taking control, but also social learning and lacking trust in governmental actions to face the pandemic as reasons for panic buying [see also (11)]. Müller and Rau (12) recently reported a link between present-bias and panic buying, while patience was associated to staying at home and avoiding crowds. On the other hand, the same study showed that all safety behaviors appear to be linked to fear, or at least COVID-19-related concern. In a systematic review, Yuen et al. (9) defined four factors that caused panic buying during a health crisis: a perception of threat and scarcity of products, fear of the unknown, panic buying as a coping behavior to deal with anxiety and to regain control, and social psychological factors.

However, Oosterhoff (13) showed that the belief that COVID-19 is a severe disease was associated with physical distancing. Self-interest was negatively associated with physical distancing, yet positively with hoarding behavior. Also, disinfection behavior was linked to greater social responsibility, while hoarding was negatively related to social responsibility and social trust. Yet, panic buying and hoarding seem to be rather related to impulsive and egoistic motives, which are particularly amplified by uncertainty [see also Chen et al. (14)]. Also, Nivette et al. (15) found that people scoring high on antisocial behaviors exhibit less compliance with public health measures such as frequent disinfection. Campos-Mercade et al. (16) showed clear associations between pro-sociality and norm-compliance. Sanitizer and face-mask use and self-isolation are self-imposed measures to stay safe oneself, but also to protect members of the community. Hence, it appears that the underlying motive may include adherence, trust toward authorities, as well as altruism.

Taken together, panic buying seems to differ from other safety behaviors. Dysfunctional safety behaviors like panic buying and hoarding maintain individual safety while neglecting possible cost for others. However, adherent safety behaviors are those that slow the spread of COVID-19, while reducing individual life-quality. They highly conform with governmentally recommended public health measures.

No research has yet directly investigated a possible distinction of these constructs with predictive or associative dynamics, although this separation would have an immense practical value. If safety behavior was one-dimensional, good policies should find an optimal tipping point at which people comply with public health measures. Yet, a conceptual distinction would propose more specific interventions to reduce panic buying and other dysfunctional behaviors on one hand, and increase adherent safety behaviors on the other hand. For instance, tailor made information or behavioral intervention campaigns could attempt to target specific risk groups which have shown increased dysfunctional safety behavior, but refuse to comply with hygiene measures. It is thus necessary to understand the psychological and environmental influences and underpinnings of both, dysfunctional safety behaviors like panic buying and adherent safety behaviors like mask wearing.

The present study assesses whether adherent and dysfunctional safety behaviors actually share the same correlates, or whether they are embedded into very different behavioral, attitudinal, intra-, and inter-individual contexts. Based on the literature, it is hypothesized that adherent and dysfunctional safety behaviors are two different sub-constructs, which are both correlated and particularly linked fear and anxiety, but show different demographic, psychological, behavioral and contextual correlates. This would suggest that political interventions by governmental authorities should very specifically aim at certain groups of individuals and adapt interventions in accordance to their motives in order to promote adherent while preventing dysfunctional safety behavior at the same time.

## Methods

### Data Collection and Participants

Data collection was performed from March 10 to May 4, 2020 in a Germany-wide online survey. During this time, Germany underwent unprecedentedly rapid changes in regulations of public space and personal freedom. For instance, schools and borders were closed and public gatherings were prohibited. Face-mask wearing became obligatory in public and commercial locations. Due to the initial success in curbing the infection rate, the German government enacted the reopening of schools, day-care centers and most commercial spaces on May 4.

Participant recruitment took place via radio, TV, social media, and newspaper. In detail, the study was announced and the respective online link to the survey was explicitly stated twice in the regional public German radio (Westdetuscher Rundfunk (WDR) 2, Lokalzeit Ruhr) on March, 12. An interview with the whole research team was shown on TV in the local public news (WDR 1, Lokalzeit Ruhr) on March, 18. Then, the head of research was interviewed by Laura Wontorra, a German TV show moderator and influencer on Instagram on April, 10. Last, the study was mentioned and its link was posted along with an interview with the head of the Clinic for Psychosomatic Medicine and Psychotherapy in the local public newspaper [Westdeutsche Allgemeine Zeitung (WAZ)] on April, 25. Of course, the link was also posted alongside a short statement on the study in health related groups in other social media platforms such as Facebook and Whatsapp.

Of 16,380 participants who commenced the survey, 15,308 completed it (completion rate = 81.02%). Due to an additional missing value, 15,307 participants will be considered for the following analyses. Informed consent was given by all participants. The study was conducted in accordance with the Ethics Committee of the Essen Medical Faculty (20-9307-BO).

### Material

Participants were asked about basic demographics, their reactions and attitudes toward the COVID-19-virus, as well as their mental health and personality. Demographic information included questions on gender (male, female or other), age in categories, education (university degree, high school diploma, secondary school degree, no secondary school degree and other form of education), current occupational status (clustered into unemployed, physician, nurse, public service – police, firefighting and paramedic), and size of the community the participants live in (metropolis with >100,000 inhabitants, medium-sized city with 100,000–20,000 inhabitants, small town with 5,000 to 20,000 inhabitants and rural community with below 5,000 inhabitants). To evaluate participants' personal hazard in case of a COVID-19 infection, the survey also assessed the presence of a high-risk morbidity for a severe course of COVID-19 (diabetes, chronic heart disease, hypertension, and chronic pulmonary disease).

The feeling of safety is of particular importance in times of crises. Safety depends on trust in authorities, subjective transparency, and knowledge. Accordingly, two self-generated scales were added measuring the trust in governmental actions to face COVID-19 (3 items, 7-point Likert-scaled) and the subjective level of information of the participants (4 items, 7-point Likert scaled; see Supplementary Material). Moreover, one item assessed COVID-19-related fear.

Safety behavior (8 items, 7-point Likert-scaled) was separated into two dimensions based an oblique factorial analysis (see Supplementary Material) – *adherent* and *dysfunctional safety behavior* including behaviors like hand-washing or physical distancing, and hoarding hygiene products or canned groceries, respectively. Cronbach's α for the scales revealed reasonable internal consistency of α = 0.65 for *trust in governmental actions* and α = 0.80 for *subjective level of information* (correlation between functional and dysfunctional safety behavior: *r* = 0.38).

It is a robust finding that media exposition might drive fear [(17–19); see also (20)]. Thus, the survey assessed which medium the participants use to remain up to date on the current happenings during the COVID-19-crisis. Single binary items (yes vs. no) were presented for information via TV, digital media, newspapers, social networks, radio, websites from public bodies, friends and family, or physicians.

The current mental health status was measured using the Patient Health Questionnaire (PHQ-2; 2 items, 4-point Likert-scaled) for depressive symptoms (21, 22) and the General Anxiety Disorder (GAD-7; 7 items, 4-point Likert-scaled) for generalized anxiety (23, 24). The survey further included the Locus of Control for its relevance in risk perception and safety behavior (25, 26), as well as the big−5 personality traits for their centrality in human behavior in general and their associations with psychopathology [Rosenström et al. (27)], measured using the Big Five Inventory (BFI-10; 10 items, 5-point Likert-scaled).

### Data Analysis

First, an oblique factorial analysis was performed to verify the two dimensions of safety behavior. Following, internal consistencies were tested for all scales. Then, all demographic, psychometric and COVID-19-related characteristics were regressed on *adherent* and *dysfunctional safety behavior*. This approach was chosen to reduce potential confounding of raw associations and take into account the contribution of other variables. Regression coefficients were treatment-coded. Yet, variable-wise F-tests are reported to illustrate each variable's overall importance. Variables were generally z-standardized to avoid multi-collinearity. Still, multi-collinearity was assessed using variance inflation factors with a criterion of 5. It was assumed that normality of residuals leaves estimates largely unbiased at large sample sizes such as the present (28). The assumption of homoskedasticity was tested using Breusch-Pagan-Tests. When homoscedasticity was violated, heteroscedasticity-robust regressions were supplemented to ensure that the results were equivalent [using the HC3 command from the R package sandwich, see also (29)].

Marginal effects are reported in the Supplementary Material. For an adequate interpretation of regression results at such high sample sizes, 95%-confidence intervals of regression weights, and effect sizes of marginal effects are reported in addition to *p*-values (30, 31). Furthermore, an effect size of <0.1 for group-wise comparisons was considered irrelevant, even if the *p*-value was below 0.05.

To find a small f^2^ of 0.02 (32) in a comparison between the actual regression model and a null model with a power of 0.99, about 3,000 participants are necessary. Given criterion of standardized regression coefficients being equal or larger than 0.1 for a meaningful interpretation, a simulated power analysis reveals that around 8,000 participants are necessary to reach a power of 0.99. Hence, the analysis is very well powered.

## Results

Table 1 shows the aggregated characteristics of the sample.

**Table 1:** Demographic information (gender, age, education, occupation, area of residence, and health status) of the study sample.

	**Overall (%)**
**N**	**15,308**
**Gender**	
Female	10,824 (70.7)
Male	4,433 (29.0)
Other	51 (0.3)
**Age (%)**	
18–24 years	2,127 (13.9)
25–34 years	3,796 (24.8)
35–44 years	3,515 (23.0)
45–54 years	2,902 (19.0)
55–64 years	2,177 (14.2)
65–74 years	670 (4.4)
above 75 years	121 (0.8)
**Education**	
University Degree	6,544 (42.7)
High School Degree	5,002 (32.7)
Secondary School Degree (Realschule)	2,791 (18.2)
First School Degree (Hauptschule)	665 (4.3)
No School Degree	48 (0.3)
Other	258 (1.7)
**Occupation**	
Unemployed	1,566 (10.2)
Physician	553 (3.6)
Nursing staff	1,682 (11.0)
Police/Firefighting/Paramedic	346 (2.3)
Student	1,987 (13.0)
Other	9,173 (59.9)
**Area**	
Large City (>100,000 inhabitants)	8,525 (55.7)
Medium-sized city (>20,000 inhabitants)	3,453 (22.6)
Small town (>5,000 inhabitants)	1,690 (11.0)
Province area (<5,000 inhabitants)	1,640 (10.7)
Risk disease (diabetes, blood pressure, cardiovascular disease, chronic pulmonic disease)	11,922 (77.9)
Mental illness	2,006 (13.1)

*Percent values in parentheses are relative to the total N = 15,308*.

To define which features were predictive of the two safety behavior dimensions, all variables, including demographic, behavioral, trait- and attitudinal variables were regressed on *adherent* and *dysfunctional safety behavior*. In both models, the assumption of homoscedasticity did not apply (Breusch-Pagan test: *p* < 0.001). Results of a heteroscedasticity-robust regression, however, yield almost identical results to the ordinary least squares regression (see Supplementary Material). None of the predictors showed a critical multi-collinearity with variance inflation factors above 5. Treatment-coded regression parameters are displayed in Table 2.

**Table 2:** Regression coefficients, 95%-confidence intervals, and *p*-values for all predictors of the regression analysis with either *adherent safety behavior* or *dysfunctional safety behavior* as dependent variables.

	**Adherent safety behavior**			**Dysfunctional safety behavior**		
**Predictors**	**Estimates**	**CI**	**p**	**Estimates**	**CI**	**p**
(Intercept)	−0.24	−0.30 to −0.18	<0.001	−0.18	−0.25 to −0.11	<0.001
Male	−0.04	−0.06 to −0.01	0.019	0.01	−0.02 to 0.05	0.456
Other Gender	−0.23	−0.44 to −0.01	0,037	0.08	−0.16 to 0.33	0.495
Dysfunctional safety behavior	0.21	0.19 to 0.22	<0.001			
Fear of COVID19	0.38	0.37 to 0.40	<0.001	0.19	0.17 to 0.21	<0.001
25–34 years	−0.04	−0.09 to 0.01	0.137	0.06	0.00 to 0.11	0.046
35–44 years	0.00	−0.05 to 0.05	0.968	0.22	0.16 to 0.28	<0.001
45–54 years	0.01	−0.04 to 0.07	0.628	0.19	0.13 to 0.25	<0.001
55–64 years	0.07	0.01 to 0.13	0.022	0.14	0.07 to 0.21	<0.001
65–74 years	0.05	−0.03 to 0.13	0.240	0.25	0.16 to 0.34	<0.001
+75 years	0.10	−0.05 to 0.25	0.213	0.35	0.17 to 0.52	<0.001
High School Degree	0.00	−0.03 to 0.03	0.939	−0.03	−0.06 to 0.01	0.135
Secondary School Degree (Realschule)	0.02	−0.02 to 0.06	0.319	−0.05	−0.09 to −0.00	0.029
First School Degree (Hauptschule)	0.03	−0.03 to 0.10	0.309	−0.06	−0.13 to 0.02	0.129
No School Degree	−0.03	−0.25 to 0.19	0.766	−0.07	−0.32 to 0.19	0.606
Other	0.00	−0.09 to 0.10	0.96	0.00	−0.11 to 0.11	0.943
Unemployed	0.06	0.01 to 0.10	0.014	0.04	−0.02 to 0.09	0.179
Physician	−0.13	−0.20 to −0.06	<0.001	0.04	−0.03 to 0.12	0.265
Nursing staff	−0.07	−0.12 to −0.03	0.001	−0.01	−0.06 to 0.04	0.643
Police/Firefighting/Paramedic	−0.20	−0.28 to −0.11	<0.001	0.01	−0.08 to 0.11	0.773
Student	−0.06	−0.11 to −0.02	0.010	−0.05	−0.11 to 0.01	0.083
Medium-sized city (>20,000)	0.08	0.04 to 0.11	<0.001	0.03	−0.00 to 0.07	0.090
Small town (>5,000)	0.15	0.11 to 0.19	<0.001	−0.01	−0.05 to 0.04	0.747
Rural area (<5,000)	0.19	0.15 to 0.23	<0.001	−0.04	−0.09 to 0.01	0.09
Yes	0.08	0.04 to 0.12	<0.001	−0.18	−0.22 to−0.13	<0.001
Yes	−0.02	−0.05 to 0.01	0.158	0.04	0.00 to 0.08	0.033
Generalized Anxiety (GAD-7)	0.07	0.05 to 0.09	<0.001	0.12	0.09 to 0.14	<0.001
Depressive Symptoms (PHQ-2)	0.02	0.00 to 0.04	0.048	−0.02	−0.04 to 0.00	0.066
Trust in governmental actions	0.16	0.15 to 0.18	<0.001	−0.08	−0.10 to −0.06	<0.001
Subjective level of information	0.09	0.07 to 0.10	<0.001	−0.04	−0.06 to −0.03	<0.001
External Locus of Control	0.03	0.02 to 0.04	<0.001	−0.04	−0.06 to −0.03	<0.001
Internal Locus of Control	−0.02	−0.03 to −0.00	0.011	0.02	−0.00 to 0.03	0.051
TV	0.14	0.12 to 0.17	<0.001	0.06	0.03 to 0.09	<0.001
Websites of public institutions	0.14	0.11 to 0.17	<0.001	−0.02	−0.05 to 0.02	0.311
Radio	−0.03	−0.06 to −0.01	0.009	−0.02	−0.05 to 0.01	0.106
Friends and acquiantances	−0.05	−0.09 to −0.02	0.003	0.10	0.06 to 0.14	<0.001
Physicians	0.00	−0.03 to 0.03	0.960	−0.01	−0.04 to 0.03	0.746
Social Networks	0.01	−0.02 to 0.04	0.439	0.02	−0.01 to 0.05	0.198
Digital Media	0.06	0.03 to 0.08	<0.001	0.03	0.00 to 0.06	0.044
Newspapers	−0.02	−0.05 to 0.00	0,096	0.05	0.02 to 0.08	0.001
BFI—Agreeableness	0.01	−0.00 to 0.02	0,123	−0.06	−0.07 to −0.04	<0.001
BFI—Neuroticism	−0.03	−0.05 to −0.02	<0.001	0.01	−0.00 to 0.03	0.146
BFI—Openness	0.02	0.01 to 0.04	<0.001	−0.01	−0.02 to 0.01	0.295
BFI—Extraversion	−0.03	−0.04 to −0.01	<0.001	0.02	0.00 to 0.03	0.017
BFI—Conscientiousness	0.02	0.01 to 0.04	<0.001	−0.03	−0.05 to −0.02	<0.001
Adherent Safety Behavior				0.27	0.25 to 0.29	<0.001
Observations	15,307	15,307				
R^2^/R^2^ adjusted	0.411/0.409	0.228/0.226				

**Figure 1:**
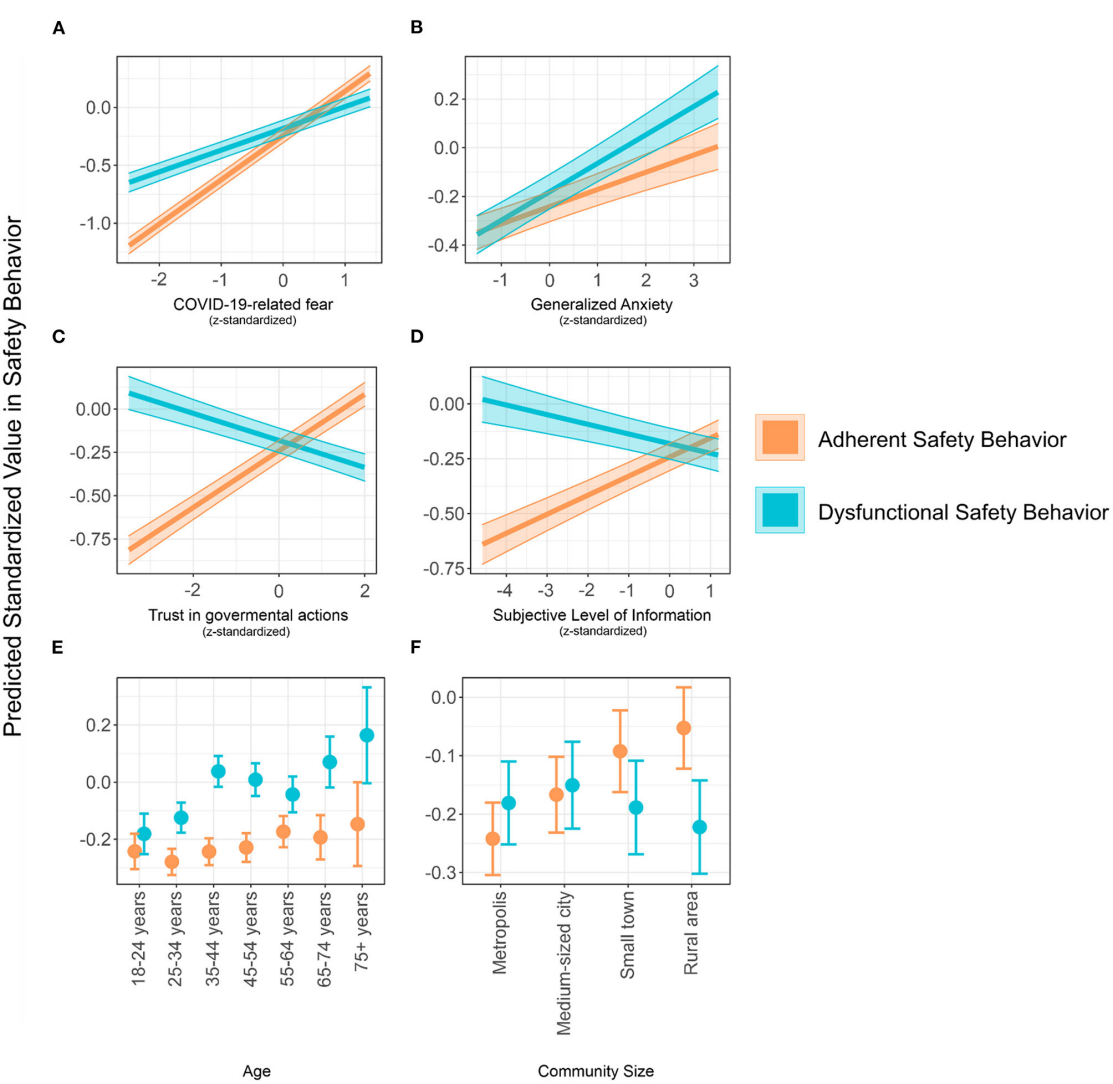
Conditional effects of regression models for the regressors COVID-19-related fear, generalized anxiety, trust in governmental actions, subjective level of information, age, and community size on either adherent or dysfunctional safety behavior. All continuous variables were z-standardized for comparability. Thick lines [panels **(A)**–**(D)**] and points [panels **(E)** and **(F)**] represent means. Error bars (for points) and colored areas (for lines) represent 95%-confidence intervals. Note: Generalized anxiety was measured by the GAD-7 (7 items, 4-point-Likert scaled), COVID-19-related fear (1 item), adherent and dysfunctional safety behavior, trust in governmental actions (4 items), and subjective level of information (4 items) were measured using self-generated Likert-type items on a 7-point-Likert scale.

The regression estimates and the marginal effects revealed similar, as well as divergent correlates of *adherent* and *dysfunctional safety behavior*. Figure 1 illustrates the marginal effects for the most pronounced differences in the regression models. *COVID-19-related fear* is positively associated with both, *adherent* and *dysfunctional safety behavior* (*F*-test in the *adherent safety behavior* model: *F*_(1, 15262)_ = 2673.15, *p* < 0.001, *F*-test in the *dysfunctional safety behavior* model: *F*_(1, 15262)_ = 430.53, *p* < 0.001, see Figure 1A). Yet, this association was more pronounced in the model predicting *adherent safety behavior* with non-overlapping confidence intervals. On the other hand, while *generalized anxiety* showed a positive relationship with both safety behaviors [*F*_(1, 15262)_ = 47.70, *p* < 0.001, for *adherent* and *F*_(1, 15262)_ = 99.82, *p* < 0.001, for *dysfunctional safety behavior*], the association with *dysfunctional safety behavior* appeared to be stronger (see Figure 1B) even though confidence intervals slightly overlapped (see Table 2).

*Trust in governmental actions* to face COVID-19 showed a strong positive relationship with *adherent safety behavior* [*F*_(1, 15262)_ = 442.43, *p* < 0.001], but a negative relationship with *dysfunctional safety behavior* [*F*_(1, 15262)_ = 75.33, *p* < 0.001, see Figure 1C]. Similarly, the *subjective level of information* was positively related to *adherent safety behavior* [*F*_(1, 15262)_ = 136.28, *p* < 0.001], but showed a negative association with *dysfunctional safety behavior* [*F*_(1, 15262)_ = 26.51, *p* < 0.001, see Figure 1D].

Age was differently associated with both safety behaviors. Although there was an upward trend in *dysfunctional safety behavior* across age [*F*_(6, 15262)_ = 15.59, *p* < 0.001], only small differences were found when predicting *adherent safety behavior* [*F*_(6, 15262)_ = 3.87, *p* = 0.001]. More specific, *dysfunctional safety behavior* increased for aged higher than 34 (see Figure 1E and Supplementary Material).

Similarly, respondents from smaller-sized communities showed more *adherent safety behavior* [*F*_(3, 15262)_ = 38.37, *p* < 0.001]. There was a gradient from metropoles to rural communities. Only the difference between small towns and rural communities was not significant (see Supplementary Material). Such a gradient did not emerge for *dysfunctional safety behavior* [*F*_(3, 15262)_ = 2.58, *p* = 0.052, see Figure 1F and Supplementary Material for marginal effects].

Pronounced differences also occurred across professional groups in predicting *adherent safety behavior* [*F*_(1, 15262)_ = 10.86, *p* < 0.001]. Predominantly, physicians, nursing staff, and people working as paramedics, firefighters and policemen showed less *dysfunctional safety behavior* than people having other occupations. Likewise, people indicating current unemployment showed more *dysfunctional safety behavior* than the other groups, except the group with other occupations (see Table 2 and Supplementary Material).

Respondents who indicated to watch TV and visit websites of public institutions to stay informed on COVID-19 showed more *adherent safety behavior* (all *F*-values > 98, *p* < 0.001), while respondents receiving information from acquaintances showed more *dysfunctional safety behavior* [*F*_(1, 15262)_ = 14.32, *p* < 0.001]. Finally, the presence of a mental disease predicted *adherent safety behavior* positively [*F*_(1, 15262)_ = 13.89, *p* < 0.001], but *dysfunctional safety behavior* negatively [*F*_(1, 15262)_ = 53.03, *p* < 0.001].

## Discussion

The COVID-19 pandemic challenged individual lives and still do to this date. While individuals were obliged to isolate themselves and showing correct hygiene behavior, others hoarded toilet paper and groceries. These *adherent* and *dysfunctional safety behaviors* may be manifestations of different motive structures and contexts. The necessary question arises, how governmental authorities can encouraged *adherent safety behavior* while attenuating *dysfunctional safety behavior*.

To investigate this highly relevant question, we hypothesized that both types of safety behaviors are associated with different set of features in terms of socio-demographics, psychopathology, personality, and COVID-19-specific attitudes. We were able to collect the largest data sample in Germany and one of the largest worldwide on the impact of COVID-19 on mental health. We specifically asked for *adherent* and *dysfunctional safety behaviors* during the COVID-19 pandemic. Despite the exploding amount of literature on the current pandemic, no other study has yet been able to report a comparable dataset profoundly investigating distinct safety behaviors in times of COVID-19 in such a detailed manner. In line with the hypothesis, the current results show that adherent and dysfunctional safety behaviors mostly differed in some of their correlates and even revealed opposite associative directionalities with others. However, some correlates were similar.

Certainly, fear and anxiety are to some degree causal for safety-oriented and preventive behaviors. Here, *COVID-19-related fear*, as well as *generalized anxiety*, separately showed positive associations with both safety behaviors. It is important to keep in mind that regression parameters are already conditioned on each other: fear and anxiety-related estimates represent the isolated contribution of each of these dimensions of safety behavior. Also, recent literature suggests a distinction between the fear of COVID-19 and anxiety (2, 5, 6, 33, 34). Importantly, the link between *COVID-19-related fear* and *adherent safety behavior* appears to be more pronounced than the link between *COVID-19-related fear* and *dysfunctional safety behavior*. A reverse pattern is observed for *generalized anxiety* – although the confidence intervals slightly overlap: the regression coefficient for *generalized anxiety* is steeper in *dysfunctional safety behavior* compared to the *adherent safety behavior*. These findings tentatively suggest that *dysfunctional safety behavior* originates from a more omnipresent feeling of threat, while *adherent safety behavior* results from a direct concern about COVID-19. Even more broadly, *dysfunctional safety behavior* could be a rather egoistic response to the feeling of overall threat. Indeed, the link between the feeling of threat, stress, and selfish behavior is being discussed for over a century now. Since Cannon (35) defined the concept of fight-or-flight, it is still found up to this point that people under acute and chronic stress incline toward less altruism, less moral decision making, and more egoistic choices (36–39). Furthermore, during the initial course of the COVID-19 pandemic, many communities suffered from shortages of goods due to previous panic-buying [see, e.g., (40)]. The perception of the risk of material deprivation may have been amplified by the subjective feeling of stress (2, 41). Thus, d*ysfunctional safety behavior may* neutralize the subjective feeling of threat by ensuring long-term material security.

The finding that *adherent safety behavior*, but not *dysfunctional safety behavior* is related to the *subjective feeling of information* and *trust in governmental intervention* fits well to the fact that *adherent safety behavior* is also rather related to fear and concern about COVID-19. *Adherent safety behavior* could arise as a product of the person's engagement with the pandemic, which would lead to overall *higher levels of information*, higher *trust in governmental actions* (perhaps even due to a higher level of information), and overall higher levels of concern. Positive associations between *adherent safety behavior* and media consumption for the sake of staying informed on COVID-19 further support the argument that more personal engagement with the COVID-19 pandemic results in more *adherent safety behavior*. Media consumption may spark fear itself, but could also function as a reassuring safety behavior itself. More engaged individuals would more likely attempt to remain up to date on recent developments to evaluate risks and regain a feeling of control. This idea is in line with the negative correlation between generalized anxiety and subjective levels of information regarding COVID-19 [(42), but see also (19)]. Again, there is some evidence that hints toward a difference in quality between *COVID-19-related fear* and *generalized anxiety* during the COVID-19 pandemic (5, 33, 34). On the other side, we only find meaningful associations between acquiring information on COVID-19 from acquaintances and *dysfunctional safety behavior*.

In the data, further differences in *adherent* and *dysfunctional safety behavior* occur between age groups. While respondents' age hardly shows any association with *adherent safety behavior*, older respondents (i.e., older than 34) indicate to engage in more *dysfunctional safety behavior*. Such increase in dysfunctional safety behavior might reflect an age-related feeling of threat by the virus [see (2) for a detailed insight of the distribution of fear across age groups]: from early on, it has been evident that people of higher age have an increased likelihood to suffer from an unfavorable course of COVID-19, which could eventually result in death (43). Thus, in anticipation of even longer self-isolation in case of infection than in younger people, preparation seems legitimate. Also, elderly are oftentimes less mobile than younger people and lockdowns make longer trips to grocery shops even more difficult. Finally, the pandemic could have cemented already pre-existing consumption styles with elderly individuals tending to buy more of some hygiene products (44).

A rather unexpected finding is that inhabitants of metropoles exhibit less adherent safety behavior than inhabitants of small towns and rural dwellers, a pattern which is not present in dysfunctional safety behavior. Here, pragmatic reasons might contribute the most: rural dwellers could simply have less difficulties to avoid crowds. Perhaps their decision threshold to even use public transit or travel around is also higher due to more autarky. Otherwise put, people living in large cities are more reliant on public transit, and partly, avoidance of crowds is hardly possible. Furthermore, Peters (45) describes the countryside as more vulnerable to potential COVID-19 outbreaks due to a lack of health services. Causes for an increased dysfunctional safety behavior could be manifold. Yet again, it is important to keep in mind that such differences cannot be explained by direct confounding via e.g., occupation or age. These variables have been conditioned upon in the regression model.

Summarizing, the presented results hint toward a differential associative, and thus contextual, embedding of adherent and dysfunctional safety behavior. Both behaviors appear to be independent of each other, which makes a differentiation theoretically and practically reasonable. People with high levels of adherent safety behavior show higher levels of authoritative trust and subjective information levels. They indicate more specific fear of COVID-19 and seem to gather more information via public news channels. Thus, adherent safety behavior could be promoted by increasing governmental responsibility, medial education, and by inducing realistic highly specialized and justified respect of a possible infection with COVID-19. Contrary to that, generalized anxiety is rather associated with panic buying and other dysfunctional safety behaviors. Accordingly, the present data suggest that governmental actions and COVID-19-specific elucidation campaigns should not target the people's general fears triggering dysfunctional safety behavior. They should rather very carefully provide profound information about virus-specific risks and possible protective countermeasures aiming for adherent safety behavior.

### Limitations

The current study has been among the few that captured the atmospheric picture during the acute period of the first COVID-19 wave in Germany. Accordingly, the study investigated the largest dataset in Germany to our knowledge and is thus of high importance for the understanding of the pandemic's impact on mental health. However, the rapid reaction to the pandemic, related quickly-evolving political decisions, and the individuals' reactions naturally comes at some negligible methodological costs.

First, and most importantly, it has to be kept in mind that all data presented here was collected via an online survey, which holds several limitations. For once, there is absolutely no way to control the participants' response rate causing the risk of a participant bias. Thus, more anxious people or those suffering from more risk factors may have responded preferentially to the survey. These points, of course, may hamper the generalizability of the present sample.

Second, at the time of the initial COVID-19 outbreak in Germany, no validated instruments were available to assess fear of COVID-19. The first questionnaire assessing COVID-19-related fear was presented after the survey had already been launched (33). The Preventive COVID-19 Behavior Scale, an instrument to measure safety behaviors [PCV-19BS, see (33, 46)], was based on recommendations by the WHO in April 2020 (47). Due to this fact, some of the scales of the survey were self-generated and COVID-19-related fear was furthermore measured by one single item.

Last, the data were collected from March 10 to May 4, 2020 and thus refer to the very first early stage of the pandemic in which people were most anxious and overstrained. By now, however, the public and governments may have adapted to the situation, which could reduce the study's relevance. However, data during just this time is rare and may thus be of special importance for socio-psychological research, even after the vulnerable phase itself. Furthermore, the second wave in Germany including a second lock-down with closed cinemas, pubs, and even boarder is happening right now in December 2020. Likewise, people begin to show panic buying behavior again, which again highlights the current data's impact.

Although some of these limitations cannot be retrospectively improved, the large sample of the current data set provides a strong variability that may legitimate an interpret a generalization and interpretation. Furthermore, safety behavior, especially during a worldwide pandemic, has not yet been investigated in comparable detail and magnitude. Apart from that, the scales used to measure *adherent* and *dysfunctional safety behavior* show decent psychometric properties (see Supplementary Material). Certainly, selection bias could play a role due to a relatively large proportion of participants, e.g., living in metropoles or pursuing medical professions. Again, a regression analysis is usually capable of partializing out such influences if considered in the model. Still, sources of confounding can be manifold [see, e.g., (48)].

### Conclusion

The present results are the first and due to data's sample size to our knowledge the most reliable in Germany to point toward two different sub-constructs of safety behavior during COVID-19. While the people's trust in governmental actions leads to adherent safety behaviors like mask wearing, anxiety may trigger panic buying and possibly increase the threshold for other-regarding welfare. These results should affect future political awareness campaigns and interventions. Especially at the present time when infection rates are raising again, political leaders now have the ability to use this data to promote preventive action and thereby avoid the further spread of COVID-19 without unfavorable backfiring.

## Data Availability Statement

The raw data supporting the conclusions of this article will be made available by the authors, without undue reservation.

## Ethics Statement

The studies involving human participants were reviewed and approved by Ethics Committee of the Essen Medical Faculty (20-9307-BO). Written informed consent for participation was not required for this study in accordance with the national legislation and the institutional requirements.

## Author Contributions

BW and AS: planning, study design, data collection, data analysis and interpretation, and manuscript writing. ND, VM, MF, and HK: data interpretation and editing. E-MS, MT, and AB: planning, study design, and supervision. All authors contributed to the article and approved the submitted version.

## Conflict of Interest

The authors declare that the research was conducted in the absence of any commercial or financial relationships that could be construed as a potential conflict of interest.
