# Supplementary material for: Different Correlates of COVID-19-Related Adherent and Dysfunctional Safety Behavior
Source: Front Public Health. 2021 Jan 13;8:625664. doi: 10.3389/fpubh.2020.625664 (PMC7838460; doi:10.3389/fpubh.2020.625664)
Supplement: Supplementary file 1 [file Data_Sheet_1.docx]

**Supplemental Online Material**

**“Different correlates of COVID-19-related adherent and dysfunctional safety behavior.”**

**Weismüller et al. (2020)**

**Supplemental Results**

**Check of regression assumptions**

In order to receive unbiased estimates in linear regression, normality of residuals, as well as homoscedasticity is usually considered obligatory. Yet, Schmidt and Finan (2018) report that normality of residuals matters less if sample sizes are adequately high. For the sake of completeness, we, however, still report the distribution of our two models’ residuals (see supplemental figure 1). Indeed, in the model considering functional safety behavior as a dependent variable, the residuals are slightly left-skewed. Our second model – considering dysfunctional safety behavior – residuals are right-skewed. Again, given the results by Schmidt and Finan (2018), we do not place much interpretational weight on these slight deviations from normality. Violations of homoscedasticity can, however, significantly bias statistical inference. Indeed, Breusch-Pagan tests for both models indicate a violation (both p < .001). We, hence, report results from heteroscedasticity robust models as performed by the sandwich R package (see also Zelleis, 2004) in supplemental table. 95%-Confidence intervals do not heavily differ from the standard OLS-model presented in the main manuscript.

**Supplemental Figure 1**


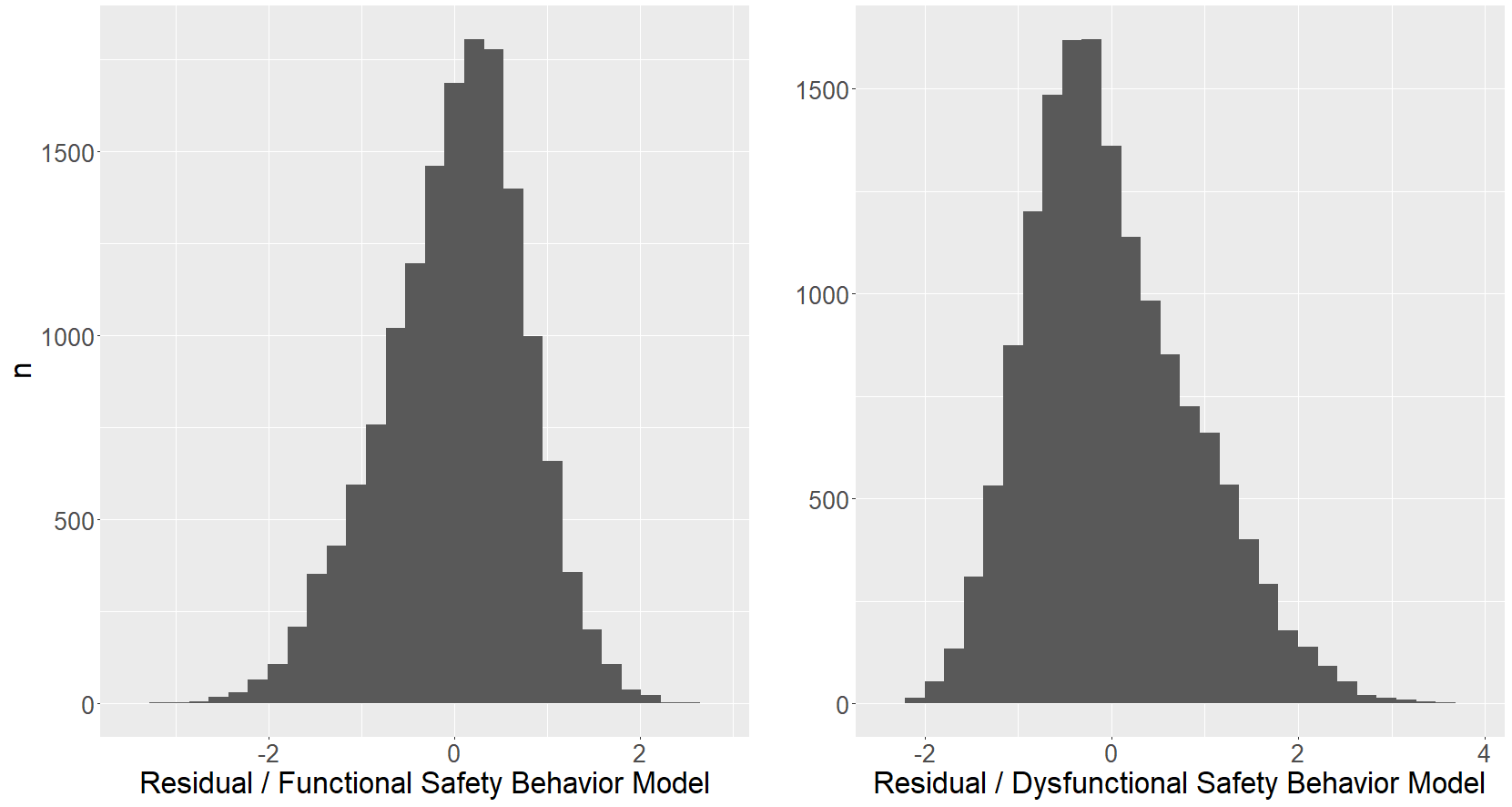


*Histograms of residuals for the model including functional (left) and dysfunctional safety behavior (right).* Indeed, residuals appear slightly skewed in both cases, but in general, such violation is negligible with large sample sizes (see Schmidt & Finan, 2018).

**Conditional Effects**

Our manuscript presents two linear regression models to assess the effect of a variety of variables on functional and dysfunctional safety behavior. Treatment-coded coefficients are presented, as well as variable wise F-tests (see supplemental table 2 for full summary). For the most important categorical predictors, we further provide marginal effects with Tukey-corrected a-errors in tables 3a-x to quantify the extent of difference between each levels.

**Self-generated questions**

Four of the scales presented in the data were self-generated: Functional and dysfunctional safety behavior, as well as the scale subjective level of information and trust in governmental actions. Furthermore, one question asked for COVID-19 related fear. Below, the reader is provided English translations of items.

*Functional Safety Behavior*

I wash / disinfect my hands more often

I increasingly avoid public places / events

I increasingly avoid public transit (subway, tram, bus train)

I have changed my trip / vacation plans or I would change them if I had planned a vacation / trip.

*Dysfunctional Safety Behavior*

I have bought larger quantities of basic food (flour, sugar, noodles, rice, and canned food) or will buy more in the near future.

I have bought larger quantities of hand disinfection/soap/similar or will buy more in the near future.

I have bought larger quantities of toilet/hygiene articles or will buy more in the near future

I have become more selfish in my behavior.

*Subjective Level of Information*

I feel informed about COVID-19

I feel informed about measures to avoid an infection with COVID-19

I understand the health authorities’ advice regarding COVID-19

*Trust in governmental actions*

1. I think all government measures are being taken to combat COVID-19
2. I have confidence in the governmental system in Germany
3. I think Germany is well prepared to face COVID-19
4. I believe that political actions against COVID-19 in Germany are exaggerated. (-)

*COVID-19-related fear*

- I worry about COVID-19.

**Factorial structure of safety behavior**

The distinction between functional and dysfunctional safety behavior is supported by a factorial analysis of the safety behavior related items. Eigenvalues above 1 generally suggest the presence of two such factors (see supplemental figure 2). However, Velicers minimum average partial test (Velicer, 1976; O’Connor, 2000) also suggests the presence of two factors.

**Supplemental Figure 2**


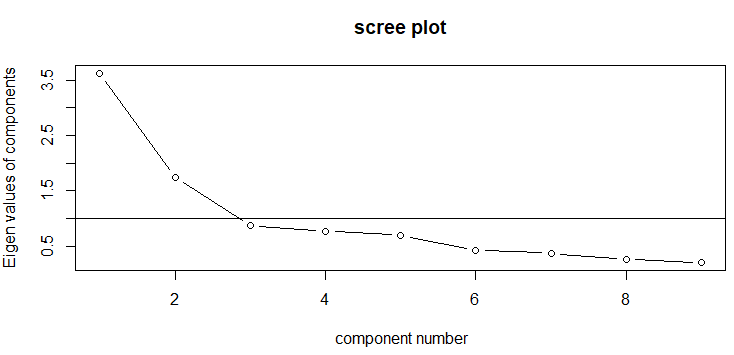


*Scree plot.*

A subsequent factor analysis with two factors using an oblimin rotation yields an appropriate fit with an RMSEA of .04 (CI = [.036 : .044] and a TLI of .99. Generally, the data is suitable for factorial analyses (the Kaiser-Meyer-Oklin Test yields an average MSA of .8 with no variable showing an MSA below .7). Factorial loadings (see supplemental table X) suggest the pattern as already presented in the section “Self-generated items”, with four items in the “functional safety behavior” and four items in the “dysfunctional safety behavior” scale. Both scales show good internal consistency with Cronbach’s a of .82 and .80 for functional and dysfunctional safety behavior, respectively.

*Supplemental table 1.* Results from robust regressions due to lacking homoscedasticity on our main models. Although 95%-CIs differ slightly, the overall pattern remains the same.

|  |  |  |  |
| --- | --- | --- | --- |
| Variable | Predictor (Treatment-coded) | Functional Safety Behavior | Dysfunctional Safety Behavior |
|  | (Intercept) | -0.24 *** | -0.18 *** |
|  |  | [-0.31, -0.18] | [-0.25, -0.11] |
| Gender | Male | -0.04 * | 0.01 |
| (ref: Female) |  | [-0.07, -0.01] | [-0.02, 0.05] |
|  | Other Gender | -0.23 | 0.08 |
|  |  | [-0.47, 0.01] | [-0.17, 0.34] |
|  | Dysfunctional Safety Behavior | 0.21 *** |  |
|  |  | [0.19, 0.22] |  |
|  | Fear of COVID19 | 0.38 *** | 0.19 *** |
|  |  | [0.37, 0.40] | [0.17, 0.21] |
| Age | 25-34 years | -0.04 | 0.06 * |
| (ref: 18-24) |  | [-0.09, 0.01] | [0.00, 0.11] |
|  | 35-44 years | -0.00 | 0.22 *** |
|  |  | [-0.06, 0.05] | [0.16, 0.28] |
|  | 45-54 years | 0.01 | 0.19 *** |
|  |  | [-0.04, 0.07] | [0.13, 0.25] |
|  | 55-64 years | 0.07 * | 0.14 *** |
|  |  | [0.01, 0.13] | [0.07, 0.20] |
|  | 65-74 years | 0.05 | 0.25 *** |
|  |  | [-0.03, 0.13] | [0.16, 0.35] |
|  | +75 years | 0.10 | 0.35 *** |
|  |  | [-0.06, 0.25] | [0.16, 0.53] |
| Education | High School Degree | 0.00 | -0.03 |
| (ref: University degree) |  | [-0.03, 0.03] | [-0.06, 0.01] |
|  | Secondary School Degree (Realschule) | 0.02 | -0.05 * |
|  |  | [-0.02, 0.06] | [-0.09, -0.00] |
|  | Secondary School Degree (Hauptschule) | 0.03 | -0.06 |
|  |  | [-0.03, 0.09] | [-0.13, 0.02] |
|  | No School Degree | -0.03 | -0.07 |
|  |  | [-0.22, 0.15] | [-0.36, 0.23] |
|  | Other | 0.00 | -0.00 |
|  |  | [-0.10, 0.10] | [-0.12, 0.11] |
| Occupation (ref. Other) | Unemployed | 0.06 ** | 0.04 |
|  |  | [0.01, 0.10] | [-0.02, 0.09] |
|  | Physician | -0.13 *** | 0.04 |
|  |  | [-0.20, -0.06] | [-0.03, 0.12] |
|  | Nursing staff | -0.07 ** | -0.01 |
|  |  | [-0.12, -0.03] | [-0.06, 0.04] |
|  | Police / Firefighting / Paramedic | -0.20 *** | 0.01 |
|  |  | [-0.29, -0.10] | [-0.08, 0.11] |
|  | Student | -0.06 * | -0.05 |
|  |  | [-0.12, -0.01] | [-0.10, 0.00] |
| Community Size | Medium-sized city (> 20 000) | 0.08 *** | 0.03 |
| (ref: Metropolis, > 100.000 |  | [0.04, 0.11] | [-0.01, 0.07] |
| inhabitants) | Small town (> 5 000) | 0.15 *** | -0.01 |
|  |  | [0.11, 0.19] | [-0.05, 0.04] |
|  | Rural area (< 5 000) | 0.19 *** | -0.04 |
|  |  | [0.15, 0.23] | [-0.09, 0.01] |
| Mental disease (ref: No) | Yes | 0.08 *** | -0.18 *** |
|  |  | [0.04, 0.12] | [-0.22, -0.13] |
| Risk disease (ref: No) | Yes | -0.02 | 0.04 * |
|  |  | [-0.05, 0.01] | [0.00, 0.08] |
|  | Generalized Anxiety (GAD-7) | 0.07 *** | 0.12 *** |
|  |  | [0.05, 0.09] | [0.09, 0.14] |
|  | Depressive Symptoms (PHQ-2) | 0.02 * | -0.02 |
|  |  | [0.00, 0.04] | [-0.04, 0.00] |
|  | Trust in governmental actions | 0.16 *** | -0.08 *** |
|  |  | [0.15, 0.18] | [-0.10, -0.06] |
|  | Subjective level of information regarding COVID19 | 0.09 *** | -0.04 *** |
|  |  | [0.07, 0.10] | [-0.06, -0.03] |
|  | External Locus of Control | 0.03 *** | -0.04 *** |
|  |  | [0.01, 0.04] | [-0.06, -0.03] |
|  | Internal Locus of Control | -0.02 * | 0.02 |
|  |  | [-0.03, -0.00] | [-0.00, 0.03] |
| Media as source of | TV | 0.14 *** | 0.06 *** |
| information regarding |  | [0.12, 0.17] | [0.03, 0.09] |
| COVID-19 | Websites of public institutions | 0.14 *** | -0.02 |
|  |  | [0.11, 0.17] | [-0.05, 0.02] |
|  | Radio | -0.03 ** | -0.02 |
|  |  | [-0.06, -0.01] | [-0.05, 0.01] |
|  | Friends and acquiantances | -0.05 ** | 0.10 *** |
|  |  | [-0.09, -0.02] | [0.06, 0.14] |
|  | Physicians | 0.00 | -0.01 |
|  |  | [-0.03, 0.03] | [-0.04, 0.03] |
|  | Social Networks | 0.01 | 0.02 |
|  |  | [-0.02, 0.04] | [-0.01, 0.05] |
|  | Quelle_digMedienja | 0.06 *** | 0.03 * |
|  |  | [0.03, 0.08] | [0.00, 0.06] |
|  | Newspapers | -0.02 | 0.05 *** |
|  |  | [-0.05, 0.00] | [0.02, 0.08] |
|  | BFI - Agreeableness | 0.01 | -0.06 *** |
|  |  | [-0.00, 0.02] | [-0.07, -0.04] |
|  | BFI - Neuroticism | -0.03 *** | 0.01 |
|  |  | [-0.05, -0.02] | [-0.00, 0.03] |
|  | BFI - Openness | 0.02 *** | -0.01 |
|  |  | [0.01, 0.04] | [-0.02, 0.01] |
|  | BFI - Extraversion | -0.03 *** | 0.02 * |
|  |  | [-0.04, -0.01] | [0.00, 0.03] |
|  | BFI - Conscientiousness | 0.02 *** | -0.03 *** |
|  |  | [0.01, 0.04] | [-0.05, -0.02] |
|  | Functional Safety Behavior |  | 0.27 *** |
|  |  |  | [0.26, 0.29] |
|  | N | 15307 | 15307 |
|  | R2 | 0.41 | 0.23 |
|  | Standard errors are heteroskedasticity robust. *** p < 0.001; ** p < 0.01; * p < 0.05. | | |
|  |  |  |  |

*Supplemental table 2*. Results of global F-tests for both main models presented in the manuscript.

|  |  |  |  |  |  |  |  |  |  |
| --- | --- | --- | --- | --- | --- | --- | --- | --- | --- |
|  | Functional Safety Behavior as dependent variable | | | |  | Dysfunctional Safety Behavior as dependent variable | | | |
| Variable | Sum.Sq | Df | F-Value | P-Value |  | Sum.Sq | Df | F-Value | P-Value |
| (Intercept) | 34.76 | 1 | 58.86 | <.001 |  | 19.37 | 1 | 25.04 | <.001 |
| Gender | 5.59 | 2 | 4.74 | 0.009 |  | 0.76 | 2 | 0.49 | 0.614 |
| Dysfunctional / Functional Safety Behavior | 540.61 | 1 | 915.43 | <.001 |  | 708.35 | 1 | 915.43 | <.001 |
| COVID-19-related fear | 1578.65 | 1 | 2673.15 | <.001 |  | 333.14 | 1 | 430.53 | <.001 |
| Age | 13.70 | 6 | 3.87 | 0.001 |  | 72.39 | 6 | 15.59 | <.001 |
| Education | 1.19 | 5 | 0.40 | 0.847 |  | 4.82 | 5 | 1.25 | 0.284 |
| Occupation | 32.06 | 5 | 10.86 | <.001 |  | 5.47 | 5 | 1.41 | 0.216 |
| Community Size | 67.97 | 3 | 38.37 | <.001 |  | 5.99 | 3 | 2.58 | 0.052 |
| Presence of Mental Disease | 8.20 | 1 | 13.89 | <.001 |  | 41.03 | 1 | 53.03 | <.001 |
| Presence of COVID-19-specific Risk Disease | 1.18 | 1 | 2.00 | 0.158 |  | 3.53 | 1 | 4.56 | 0.033 |
| GAD-7 | 28.17 | 1 | 47.70 | <.001 |  | 77.24 | 1 | 99.82 | <.001 |
| PHQ-2 | 2.31 | 1 | 3.91 | 0.048 |  | 2.62 | 1 | 3.39 | 0.066 |
| Trust in Governmental Interventions | 261.28 | 1 | 442.43 | <.001 |  | 58.29 | 1 | 75.33 | <.001 |
| Subjective level of information regarding COVID-19 | 80.48 | 1 | 136.28 | 0.000 |  | 20.52 | 1 | 26.51 | <.001 |
| Locus of Control - external | 9.79 | 1 | 16.58 | <.001 |  | 20.48 | 1 | 26.47 | <.001 |
| Locus of Control - internal | 3.85 | 1 | 6.53 | 0.011 |  | 2.96 | 1 | 3.82 | 0.051 |
| Source: TV | 61.95 | 1 | 104.89 | <.001 |  | 11.08 | 1 | 14.32 | <.001 |
| Source: Websites of Public Institutions | 58.23 | 1 | 98.60 | <.001 |  | 0.80 | 1 | 1.03 | 0.311 |
| Source: Radio | 4.01 | 1 | 6.79 | 0.009 |  | 2.02 | 1 | 2.61 | 0.106 |
| Source: Acquaintances | 5.13 | 1 | 8.69 | 0.003 |  | 19.69 | 1 | 25.44 | <.001 |
| Source: Physicians | 0.00 | 1 | 0.00 | 0.960 |  | 0.08 | 1 | 0.10 | 0.746 |
| Source: Social Networks | 0.35 | 1 | 0.60 | 0.439 |  | 1.28 | 1 | 1.65 | 0.198 |
| Source: Digital Media | 10.47 | 1 | 17.73 | <.001 |  | 3.14 | 1 | 4.06 | 0.044 |
| Source: Newspaper | 1.64 | 1 | 2.77 | 0.096 |  | 8.86 | 1 | 11.46 | 0.001 |
| BFI - Agreeableness | 1.41 | 1 | 2.38 | 0.123 |  | 47.73 | 1 | 61.69 | <.001 |
| BFI - Neuroticism | 9.10 | 1 | 15.41 | <.001 |  | 1.63 | 1 | 2.11 | 0.146 |
| BFI - Openness | 8.51 | 1 | 14.41 | <.001 |  | 0.85 | 1 | 1.10 | 0.295 |
| BFI - Extraversion | 9.08 | 1 | 15.37 | <.001 |  | 4.39 | 1 | 5.68 | 0.017 |
| BFI - Concientiousness | 7.16 | 1 | 12.13 | <.001 |  | 12.87 | 1 | 16.63 | <.001 |
| Residuals | 9013.07 | 15262 |  |  |  | 11809.62 | 15262 |  |  |
|  |  |  |  |  |  |  |  |  |  |

| Table 3a. Simple Effects of Gender on Functional Safety Behavior | | | | | | | | |
| --- | --- | --- | --- | --- | --- | --- | --- | --- |
| Contrast | Estimate | Standard Error | df | T-Value | p-Value | 95%-CI lower bound | 95%-CI upper bound | Cohen's d |
| female vs. male | 0.035 | 0.015 | 15262 | 2.354 | 0.049 | 0.000 | 0.071 | 0.046 |
| female vs. other | 0.226 | 0.108 | 15262 | 2.086 | 0.093 | -0.028 | 0.480 | 0.294 |
| male vs. other | 0.190 | 0.109 | 15262 | 1.753 | 0.186 | -0.064 | 0.445 | 0.248 |
|  |  |  |  |  |  |  |  |  |
| Table 3b. Simple Effects of Gender on Dysunctional Safety Behavior | | | | | | | | |
| Contrast | Estimate | Standard Error | df | T-Value | p-Value | 95%-CI lower bound | 95%-CI upper bound | Cohen's d |
| female vs. male | -0.013 | 0.017 | 15262 | -0.745 | 0.737 | -0.053 | 0.027 | -0.015 |
| female vs. other | -0.085 | 0.124 | 15262 | -0.682 | 0.774 | -0.375 | 0.206 | -0.096 |
| male vs. other | -0.072 | 0.124 | 15262 | -0.577 | 0.832 | -0.363 | 0.220 | -0.082 |
|  |  |  |  |  |  |  |  |  |
| Table 3c. Simple Effects of Age on Functional Safety Behavior | | | | | | | | |
| Contrast | Estimate | Standard Error | df | T-Value | p-Value | 95%-CI lower bound | 95%-CI upper bound | Cohen's d |
| 18-24 years vs. 25-34 years | 0.037 | 0.025 | 15262 | 1.486 | 0.753 | -0.036 | 0.109 | 0.048 |
| 18-24 years vs. 35-44 years | 0.001 | 0.027 | 15262 | 0.040 | 1.000 | -0.078 | 0.081 | 0.001 |
| 18-24 years vs. 45-54 years | -0.014 | 0.028 | 15262 | -0.484 | 0.999 | -0.096 | 0.069 | -0.018 |
| 18-24 years vs. 55-64 years | -0.069 | 0.030 | 15262 | -2.291 | 0.248 | -0.158 | 0.020 | -0.090 |
| 18-24 years vs. 65-74 years | -0.049 | 0.042 | 15262 | -1.176 | 0.903 | -0.172 | 0.074 | -0.064 |
| 18-24 years vs. 75+ years | -0.095 | 0.076 | 15262 | -1.245 | 0.876 | -0.321 | 0.130 | -0.124 |
| 25-34 years vs. 35-44 years | -0.036 | 0.019 | 15262 | -1.917 | 0.469 | -0.090 | 0.019 | -0.046 |
| 25-34 years vs. 45-54 years | -0.050 | 0.020 | 15262 | -2.496 | 0.161 | -0.110 | 0.009 | -0.065 |
| 25-34 years vs. 55-64 years | -0.106 | 0.023 | 15262 | -4.628 | 0.000 | -0.173 | -0.038 | -0.137 |
| 25-34 years vs. 65-74 years | -0.086 | 0.037 | 15262 | -2.346 | 0.222 | -0.193 | 0.022 | -0.111 |
| 25-34 years vs. 75+ years | -0.132 | 0.074 | 15262 | -1.786 | 0.557 | -0.350 | 0.086 | -0.172 |
| 35-44 years vs. 45-54 years | -0.015 | 0.020 | 15262 | -0.751 | 0.989 | -0.072 | 0.043 | -0.019 |
| 35-44 years vs. 55-64 years | -0.070 | 0.022 | 15262 | -3.178 | 0.025 | -0.135 | -0.005 | -0.091 |
| 35-44 years vs. 65-74 years | -0.050 | 0.036 | 15262 | -1.400 | 0.802 | -0.155 | 0.055 | -0.065 |
| 35-44 years vs. 75+ years | -0.096 | 0.073 | 15262 | -1.312 | 0.847 | -0.313 | 0.120 | -0.125 |
| 45-54 years vs. 55-64 years | -0.055 | 0.022 | 15262 | -2.495 | 0.161 | -0.121 | 0.010 | -0.072 |
| 45-54 years vs. 65-74 years | -0.035 | 0.036 | 15262 | -0.993 | 0.956 | -0.140 | 0.070 | -0.046 |
| 45-54 years vs. 75+ years | -0.082 | 0.073 | 15262 | -1.115 | 0.924 | -0.297 | 0.134 | -0.106 |
| 55-64 years vs. 65-74 years | 0.020 | 0.035 | 15262 | 0.562 | 0.998 | -0.085 | 0.125 | 0.026 |
| 55-64 years vs. 75+ years | -0.026 | 0.073 | 15262 | -0.360 | 1.000 | -0.242 | 0.189 | -0.034 |
| 65-74 years vs. 75+ years | -0.046 | 0.076 | 15262 | -0.607 | 0.997 | -0.271 | 0.179 | -0.060 |
|  |  |  |  |  |  |  |  |  |
| Table 3d. Simple Effects of Age on Dysfunctional Safety Behavior | | | | | | | | |
| Contrast | Estimate | Standard Error | df | T-Value | p-Value | 95%-CI lower bound | 95%-CI upper bound | Cohen's d |
| 18-24 years vs. 25-34 years | -0.056 | 0.028 | 15262 | -1.999 | 0.415 | -0.140 | 0.027 | -0.064 |
| 18-24 years vs. 35-44 years | -0.219 | 0.031 | 15262 | -7.106 | 0.000 | -0.310 | -0.128 | -0.249 |
| 18-24 years vs. 45-54 years | -0.190 | 0.032 | 15262 | -5.902 | 0.000 | -0.284 | -0.095 | -0.216 |
| 18-24 years vs. 55-64 years | -0.138 | 0.034 | 15262 | -4.006 | 0.001 | -0.239 | -0.036 | -0.157 |
| 18-24 years vs. 65-74 years | -0.251 | 0.048 | 15262 | -5.279 | 0.000 | -0.392 | -0.111 | -0.286 |
| 18-24 years vs. 75+ years | -0.345 | 0.087 | 15262 | -3.945 | 0.002 | -0.603 | -0.087 | -0.392 |
| 25-34 years vs. 35-44 years | -0.162 | 0.021 | 15262 | -7.647 | 0.000 | -0.225 | -0.100 | -0.184 |
| 25-34 years vs. 45-54 years | -0.133 | 0.023 | 15262 | -5.776 | 0.000 | -0.201 | -0.065 | -0.151 |
| 25-34 years vs. 55-64 years | -0.081 | 0.026 | 15262 | -3.115 | 0.030 | -0.158 | -0.004 | -0.093 |
| 25-34 years vs. 65-74 years | -0.195 | 0.042 | 15262 | -4.667 | 0.000 | -0.318 | -0.072 | -0.222 |
| 25-34 years vs. 75+ years | -0.289 | 0.084 | 15262 | -3.416 | 0.011 | -0.538 | -0.040 | -0.328 |
| 35-44 years vs. 45-54 years | 0.029 | 0.022 | 15262 | 1.302 | 0.851 | -0.037 | 0.095 | 0.033 |
| 35-44 years vs. 55-64 years | 0.081 | 0.025 | 15262 | 3.208 | 0.023 | 0.007 | 0.155 | 0.092 |
| 35-44 years vs. 65-74 years | -0.033 | 0.041 | 15262 | -0.797 | 0.985 | -0.153 | 0.088 | -0.037 |
| 35-44 years vs. 75+ years | -0.126 | 0.084 | 15262 | -1.505 | 0.742 | -0.374 | 0.121 | -0.144 |
| 45-54 years vs. 55-64 years | 0.052 | 0.025 | 15262 | 2.039 | 0.390 | -0.023 | 0.127 | 0.059 |
| 45-54 years vs. 65-74 years | -0.062 | 0.041 | 15262 | -1.517 | 0.735 | -0.182 | 0.058 | -0.070 |
| 45-54 years vs. 75+ years | -0.156 | 0.084 | 15262 | -1.857 | 0.509 | -0.403 | 0.091 | -0.177 |
| 55-64 years vs. 65-74 years | -0.113 | 0.041 | 15262 | -2.795 | 0.077 | -0.233 | 0.006 | -0.129 |
| 55-64 years vs. 75+ years | -0.207 | 0.084 | 15262 | -2.481 | 0.166 | -0.454 | 0.039 | -0.236 |
| 65-74 years vs. 75+ years | -0.094 | 0.087 | 15262 | -1.075 | 0.936 | -0.351 | 0.163 | -0.107 |
|  |  |  |  |  |  |  |  |  |
| Table 3e. Simple Effects of Community Size on Functional Safety Behavior | | | | | | | | |
| Contrast | Estimate | Standard Error | df | T-Value | p-Value | 95%-CI lower bound | 95%-CI upper bound | Cohen's d |
| Metropolis vs. Medium-sized city | -0.076 | 0.016 | 15262 | -4.816 | 0 | -0.116 | -0.035 | -0.098 |
| Metropolis vs. Small town | -0.15 | 0.021 | 15262 | -7.223 | 0 | -0.203 | -0.097 | -0.195 |
| Metropolis vs. Rural area | -0.19 | 0.021 | 15262 | -8.973 | 0 | -0.244 | -0.135 | -0.247 |
| Medium-sized city vs. Small town | -0.074 | 0.023 | 15262 | -3.248 | 0.006 | -0.133 | -0.016 | -0.097 |
| Medium-sized city vs. Rural area | -0.114 | 0.023 | 15262 | -4.927 | 0 | -0.174 | -0.055 | -0.148 |
| Small town vs. Rural area | -0.04 | 0.027 | 15262 | -1.492 | 0.442 | -0.108 | 0.029 | -0.052 |
|  |  |  |  |  |  |  |  |  |
| Table 3f. Simple Effects of Community Size on Dysfunctional Safety Behavior | | | | | | | | |
| Contrast | Estimate | Standard Error | df | T-Value | p-Value | 95%-CI lower bound | 95%-CI upper bound | Cohen's d |
| Metropolis vs. Medium-sized city | -0.03 | 0.018 | 15262 | -1.693 | 0.327 | -0.077 | 0.016 | -0.035 |
| Metropolis vs. Small town | 0.008 | 0.024 | 15262 | 0.323 | 0.988 | -0.053 | 0.069 | 0.009 |
| Metropolis vs. Rural area | 0.041 | 0.024 | 15262 | 1.698 | 0.325 | -0.021 | 0.103 | 0.047 |
| Medium-sized city vs. Small town | 0.038 | 0.026 | 15262 | 1.455 | 0.465 | -0.029 | 0.105 | 0.043 |
| Medium-sized city vs. Rural area | 0.072 | 0.027 | 15262 | 2.7 | 0.035 | 0.003 | 0.14 | 0.081 |
| Small town vs. Rural area | 0.033 | 0.031 | 15262 | 1.097 | 0.692 | -0.045 | 0.112 | 0.038 |
|  |  |  |  |  |  |  |  |  |
| Table 3g. Simple Effects of Occupation on Functional Safety Behavior | | | | | | | | |
| Contrast | Estimate | Standard Error | df | T-Value | p-Value | 95%-CI lower bound | 95%-CI upper bound | Cohen's d |
| Other vs. Unemployed | -0.057 | 0.023 | 15262 | -2.448 | 0.140 | -0.123 | 0.009 | -0.074 |
| Other vs. Physician | 0.130 | 0.035 | 15262 | 3.740 | 0.003 | 0.031 | 0.229 | 0.169 |
| Other vs. Nursing Staff | 0.073 | 0.022 | 15262 | 3.400 | 0.009 | 0.012 | 0.135 | 0.095 |
| Other vs. Paramedic / Firefighting / Police | 0.195 | 0.043 | 15262 | 4.510 | 0.000 | 0.072 | 0.318 | 0.254 |
| Other vs. Student | 0.065 | 0.025 | 15262 | 2.584 | 0.101 | -0.007 | 0.136 | 0.084 |
| Unemployed vs. Physician | 0.187 | 0.041 | 15262 | 4.599 | 0.000 | 0.071 | 0.303 | 0.243 |
| Unemployed vs. Nursing Staff | 0.130 | 0.029 | 15262 | 4.418 | 0.000 | 0.046 | 0.214 | 0.169 |
| Unemployed vs. Paramedic / Firefighting / Police | 0.252 | 0.048 | 15262 | 5.243 | 0.000 | 0.115 | 0.389 | 0.328 |
| Unemployed vs. Student | 0.121 | 0.032 | 15262 | 3.792 | 0.002 | 0.030 | 0.213 | 0.158 |
| Physician vs. Nursing Staff | -0.057 | 0.040 | 15262 | -1.442 | 0.701 | -0.170 | 0.056 | -0.074 |
| Physician vs. Paramedic / Firefighting / Police | 0.065 | 0.054 | 15262 | 1.196 | 0.839 | -0.090 | 0.219 | 0.084 |
| Physician vs. Student | -0.066 | 0.042 | 15262 | -1.573 | 0.616 | -0.185 | 0.053 | -0.085 |
| Nursing Staff vs. Paramedic / Firefighting / Police | 0.122 | 0.046 | 15262 | 2.628 | 0.091 | -0.010 | 0.254 | 0.159 |
| Nursing Staff vs. Student | -0.009 | 0.030 | 15262 | -0.288 | 1.000 | -0.094 | 0.077 | -0.011 |
| Paramedic / Firefighting / Police vs. Student | -0.130 | 0.048 | 15262 | -2.739 | 0.068 | -0.266 | 0.005 | -0.170 |
|  |  |  |  |  |  |  |  |  |
| Table 3h. Simple Effects of Occupation on Dysfunctional Safety Behavior | | | | | | | | |
| Contrast | Estimate | Standard Error | df | T-Value | p-Value | 95%-CI lower bound | 95%-CI upper bound | Cohen's d |
| Other vs. Unemployed | -0.036 | 0.027 | 15262 | -1.345 | 0.760 | -0.111 | 0.040 | -0.041 |
| Other vs. Physician | -0.044 | 0.040 | 15262 | -1.114 | 0.876 | -0.158 | 0.069 | -0.051 |
| Other vs. Nursing Staff | 0.011 | 0.025 | 15262 | 0.464 | 0.997 | -0.059 | 0.082 | 0.013 |
| Other vs. Paramedic / Firefighting / Police | -0.014 | 0.050 | 15262 | -0.289 | 1.000 | -0.155 | 0.127 | -0.016 |
| Other vs. Student | 0.050 | 0.029 | 15262 | 1.733 | 0.510 | -0.032 | 0.131 | 0.056 |
| Unemployed vs. Physician | -0.009 | 0.047 | 15262 | -0.187 | 1.000 | -0.141 | 0.124 | -0.010 |
| Unemployed vs. Nursing Staff | 0.047 | 0.034 | 15262 | 1.400 | 0.727 | -0.049 | 0.143 | 0.054 |
| Unemployed vs. Paramedic / Firefighting / Police | 0.021 | 0.055 | 15262 | 0.389 | 0.999 | -0.135 | 0.178 | 0.024 |
| Unemployed vs. Student | 0.085 | 0.037 | 15262 | 2.328 | 0.183 | -0.019 | 0.190 | 0.097 |
| Physician vs. Nursing Staff | 0.056 | 0.045 | 15262 | 1.235 | 0.820 | -0.073 | 0.185 | 0.064 |
| Physician vs. Paramedic / Firefighting / Police | 0.030 | 0.062 | 15262 | 0.486 | 0.997 | -0.147 | 0.207 | 0.034 |
| Physician vs. Student | 0.094 | 0.048 | 15262 | 1.968 | 0.361 | -0.042 | 0.230 | 0.107 |
| Nursing Staff vs. Paramedic / Firefighting / Police | -0.026 | 0.053 | 15262 | -0.485 | 0.997 | -0.177 | 0.126 | -0.029 |
| Nursing Staff vs. Student | 0.038 | 0.034 | 15262 | 1.108 | 0.878 | -0.060 | 0.136 | 0.043 |
| Paramedic / Firefighting / Police vs. Student | 0.064 | 0.055 | 15262 | 1.171 | 0.851 | -0.092 | 0.219 | 0.073 |
|  |  |  |  |  |  |  |  |  |
| Table 3.: Simple Effects of use of public health websites to remain informed on Functional Safety Behavior | | | | | | | | |
| Contrast | Estimate | Standard Error | df | T-Value | p-Value | 95%-CI lower bound | 95%-CI upper bound | Cohen's d |
| No vs. Yes | -0.142 | 0.014 | 15262 | -9.930 | 0.000 | -0.170 | -0.114 | -0.185 |
|  |  |  |  |  |  |  |  |  |
| Table 3j. Simple Effects of use of public health websites to remain informed on Dysfunctional Safety Behavior | | | | | | | | |
| Contrast | Estimate | Standard Error | df | T-Value | p-Value | 95%-CI lower bound | 95%-CI upper bound | Cohen's d |
| No vs. Yes | 0.017 | 0.016 | 15262 | 1.014 | 0.311 | -0.016 | 0.049 | 0.019 |
|  |  |  |  |  |  |  |  |  |
| Table 3k. Simple Effects of use of TV to remain informed on Functional Safety Behavior | | | | | | | | |
| Contrast | Estimate | Standard Error | df | T-Value | p-Value | 95%-CI lower bound | 95%-CI upper bound | Cohen's d |
| No vs. Yes | -0.145 | 0.014 | 15262 | -10.242 | 0.000 | -0.172 | -0.117 | -0.188 |
|  |  |  |  |  |  |  |  |  |
| Table 3l. Simple Effects of use of TV to remain informed on Dysfunctional Safety Behavior | | | | | | | | |
| Contrast | Estimate | Standard Error | df | T-Value | p-Value | 95%-CI lower bound | 95%-CI upper bound | Cohen's d |
| No vs. Yes | -0.061 | 0.016 | 15262 | -3.784 | 0.000 | -0.093 | -0.030 | -0.070 |
|  |  |  |  |  |  |  |  |  |
| Table 3m. Simple Effects of Presence of a Mental Disease on Functional Safety Behavior | | | | | | | | |
| Contrast | Estimate | Standard Error | df | T-Value | p-Value | 95%-CI lower bound | 95%-CI upper bound | Cohen's d |
| No vs. Yes | -0.079 | 0.021 | 15262 | -3.726 | 0.000 | -0.120 | -0.037 | -0.102 |
|  |  |  |  |  |  |  |  |  |
| Table 3n. Simple Effects of Presence of a Mental Disease on Dysfunctional Safety Behavior | | | | | | | | |
| Contrast | Estimate | Standard Error | df | T-Value | p-Value | 95%-CI lower bound | 95%-CI upper bound | Cohen's d |
| No vs. Yes | 0.176 | 0.024 | 15262 | 7.282 | 0.000 | 0.128 | 0.223 | 0.200 |
|  |  |  |  |  |  |  |  |  |
| Table 3o. Simple Effects of talking to Acquaintances as a Source of Information on Functional Safety Behavior | | | | | | | | |
| Contrast | Estimate | Standard Error | df | T-Value | p-Value | 95%-CI lower bound | 95%-CI upper bound | Cohen's d |
| No vs. Yes | 0.051 | 0.017 | 15262 | 2.948 | 0.003 | 0.017 | 0.085 | 0.067 |
|  |  |  |  |  |  |  |  |  |
| Table 3p. Simple Effects of talking to Acquaintances as a Source of Information on Dysfunctional Safety Behavior | | | | | | | | |
| Contrast | Estimate | Standard Error | df | T-Value | p-Value | 95%-CI lower bound | 95%-CI upper bound | Cohen's d |
| No vs. Yes | -0.100 | 0.020 | 15262 | -5.044 | 0.000 | -0.139 | -0.061 | -0.114 |

*Table 4. Factorial loadings for the two safety behavior factors – functional and dysfunctional safety behavior.*

|  |  |  |
| --- | --- | --- |
| Item | Functional Safety Behavior | Dysfunctional Safety Behavior |
|  |  |  |
| I wash / disinfect my hands more often | 0.38 | 0.15 |
| I have bought larger quantities of basic food (flour, sugar, noodles, rice, and canned food) or will buy more in the near future. | 0.07 | 0.76 |
| I have bought larger quantities of hand disinfection/soap/similar or will buy more in the near future. | 0.06 | 0.78 |
| I have bought larger quantities of toilet/hygiene articles or will buy more in the near future | -0.07 | 0.91 |
| I increasingly avoid public places/ events. | 0.93 | -0.02 |
| I increasingly avoid public transit (subway, tram, bus, train). | 0.84 | 0.01 |
| I have changed my trip/ vacation plans or would change them if I had planned a vacation/trip | 0.71 | 0.02 |
| I have become more selfish in my behavior. | 0.1 | 0.32 |
| Correlation between factors = .37. RMSEA = 0.04, TLI = .99. | | |
|  |  |  |

**References**

O’Connor, B. P. (2000). SPSS and SAS programs for determining the number of components using parallel analysis and Velicer’s MAP test. *Behavior research methods, instruments, & computers*, *32*(3), 396-402.

Schmidt, A. F., & Finan, C. (2018). Linear regression and the normality assumption. *Journal of clinical epidemiology*, *98*, 146-151.

Velicer, W. F. (1976). Determining the number of components from the matrix of partial correlations. *Psychometrika*, *41*(3), 321-327.

Zeileis, A. (2004). Econometric computing with HC and HAC covariance matrix estimators.
